# Artificial intelligence for predicting depression anxiety and stress using psychometric data

**DOI:** 10.1038/s41598-025-21301-1

**Published:** 2025-10-24

**Authors:** Tamer ShamsEldin, Sarah Gaber, Shuja Ansari, Rania Elgohary, Mahmoud A. Shawky, Magdy Elbahnasawy, Mohammed Abdrabou

**Affiliations:** 1 Technical Research Center, Cairo, 11765 Egypt; 2 British University in Egypt, Cairo, 11837 Egypt; 3 James Watt School of Engineering, University of Glasgow, Glasgow, G12 8QQ Scotland, UK; 4 Department of Info. Systems, Faculty of Computer and Info. Sciences, Ain Shams University, Cairo, Egypt; 5 Faculty of Informatics & Computer Science, German International University, Cairo, Egypt; 6 The Egyptian Technical Research and Development Centre, 11618, Cairo, Egypt

**Keywords:** Artificial intelligence, Machine learning, Mental health, Support vector machine, Decision tree (DT), Random forest (RF), K-nearest neighbor (KNN), Naive Bayes (NB), Depression anxiety stress scales-42 questionnaire., Computational biology and bioinformatics, Diseases, Health care, Mathematics and computing, Psychology, Psychology

## Abstract

Mental health is a crucial aspect of overall well-being, yet it is often overlooked due to stigma and limited accessibility to care. This study investigates the ability of artificial intelligence (AI) to predict common psychological conditions, depression, anxiety, and stress, using validated psychometric data. We analyzed responses from the Depression Anxiety Stress Scales-42 (DASS-42) questionnaire, combined with demographic information, drawn from a large publicly available dataset of 39, 775 anonymized participants. Five machine learning models were evaluated: decision tree, random forest, k-nearest neighbor, naive Bayes, and support vector machine (SVM). Data preprocessing included handling missing values, demographic standardization, and validity checks. Model performance was assessed using stratified train-test splits and five-fold cross-validation. The SVM model achieved the highest accuracy (99.3% for depression, 98.9% for anxiety, 98.8% for stress). These findings highlight the potential of AI-based approaches for early mental health screening, although further clinical validation is necessary to ensure their real-world applicability.

## Introduction

Mental illness is a problem that affects mental health of people including their feelings, ways of thinking, behavior, and interactions with other people. According to world health organization (WHO), there are 12.5% person in the world live suffer a mental illness^1^, which is a ratio that must be considered carefully. Mental illness has different types such as anxiety, depression, schizophrenia, eating disorder, bipolar disorder, stress, post-traumatic stress disorder (PTSD), disruptive behavior and dissocial disorders, and neuro-developmental disorders. Beside having different degrees such as normal, mild, moderate, severe, and extremely severe^2^. It hits people with different genders, and ages with different symptoms but it leads to the same consequences where people’s life is affected negatively and may lead to suicidal thoughts. The lack of awareness of mental health may lead to severe mental illness, on the other hand, early diagnosing of mental illness helps in getting back to normal life faster easily.

Artificial intelligence (AI) has proven its use in healthcare applications in which performing tasks becomes easier and more efficient for detection and diagnosing of diseases, drugs development and predictive analysis^3^. The improvement in collecting and analyzing data from images, voice records, videos, posts, and interaction over social media platforms made AI very useful in mental healthcare. Sometimes it is hard for therapists to make effective diagnosing of certain mental illness due to different types of mental illness that share the same symptoms. As well as therapist is a human who may be affected by patient’s words and talks. On the other hand, machines have no emotions or feelings that can be affected, which makes diagnosing more efficient^2,4^.

Mental illness prediction using machine learning (ML) has gained significant attention, yet existing approaches often lack generalizability, rely on limited algorithms, or fail to integrate validated psychometric tools effectively. This study addresses three key research questions: (1) How do various ML algorithms (e.g., RF, DT, NB, KNN, SVM) compare in predicting mental illness? (2) Can a novel ML-based predictive framework improve accuracy and robustness in mental health assessment? (3) How effectively can the DASS-42 questionnaire, a standardized psychological tool, enhance model training and evaluation? While prior studies have explored ML for mental health, gaps remain in systematic algorithm comparisons, integrative frameworks, and the use of clinically validated metrics like DASS-42. Our work bridges these gaps by conducting a comprehensive review of ML techniques, proposing a multi-algorithm predictive framework, and rigorously evaluating performance using accuracy, precision, recall, and F1-score, offering a more reliable approach for clinical and research applications.

Mental illness has a clearly noticeable symptoms such as eating and sleeping problems, headaches, fatigue, mood swings, difficulty in concentration, and suicidal thoughts^5^. However, people give no attention to their feelings and neglect the signals that indicates a problem with their mental well-being^6^. The fear of society has a huge impact on person’s actions towards mental illness, where the person fears being judged or rejected by the people around him^7^.

People are victims of the misconception surrounding mental health. One of the myths of mental illness is that people suffering from mental illness has unpredictable behavior towards people communicating with, which may extend to people being afraid to communicate or work with them. Social stigma indicates the myths, misconceptions and beliefs that challenge mental health, Although patients are aware of their mental illness, they avoid seeking professional help due to stigma around mental health^8^. Some individuals may prefer digital interactions when discussing personal mental health issues due to privacy and stigma concerns^9^. Most people prefer interacting with machines rather than interacting with human when it comes talking about their private information and feelings. They believe that it’s easier to interact with machines that do not have the power to reveal their personal information or influence their lives^10^. This study relies on publicly available, anonymized data hosted on Kaggle. No direct interaction with human subjects occurred. Therefore, institutional review board (IRB) approval was not required. The dataset creators collected participant consent under their respective terms of use and privacy conditions.

### Mental health

Mental health can be defined by person’s feelings, thoughts, behavior, and his mental well-being. Mental illness is defined as mental health disorder that includes some conditions that negatively affect a person’s psychological health, which greatly affects the individual’s daily life, his job performance, and his communication with other people. Mental health is not statically stable, it fluctuates from good to bad. Also, it is not limited to prevent mental illness, but it includes improving mental health in general^11,12^. There are many types of mental disorders. In this paper, depression, anxiety, and stress disorders are considered.

- Depression: Is the most common mental illness. People may experience various symptoms such as mood symptoms, which are related to an individual’s emotions and feelings and include feeling of guilt and unworthiness, sadness, anger, low strength, and loss of interest in many activities^12^. Also, physiological symptoms which involve the physical changes that have an impact on an individual’s body and their physical health and include fatigue, low energy, loss of appetite and sleep disturbance^13,14^. In addition to cognitive symptoms that involve changes in an individual’s thoughts, vision, and beliefs about life, these includes suicidal thoughts, lack of concentration, low self-esteem, and lack of confidence^11,15^.
- Anxiety: Is a mental illness that results from thoughts and situations that make a person feel fearful and anxious. Sever anxiety leads to attacks of fear and panic, which is known as panic disorder that affects an individual’s daily life^11^. There are many symptoms for anxiety such as mood symptoms, which are related to an individual’s emotions and feelings and include feelings of anxiety, nervousness, insecurity, and danger^5^. Also, physiological symptoms which involve the physical changes that have impact on individual’s body and physical health and include breathing problems, rapid heartbeat, sweating, fatigue, irritability, and exhaustion. In addition to cognitive symptoms that involve changes in an individual’s thoughts, vision, and beliefs about life, that mainly include fear, fear of judgment, loss of control, lack of concentration, and painful thoughts^16^.
- Stress: Is a normal human response to a specific activity or event that a person experiences. An individual may experience a positive or negative effect of stress on their body and mind, positive stress is important for overall mental health, as small amount of stress can alert a person and motivate them to perform better at important events and tasks^16^. On the other hand, difficult situations lead to a high level of anxiety, which leads to a negative psychological stress, which affects the individual’s daily life, and excessive stress leads to mental illness. There are many symptoms for stress, such as mood symptoms that can appear as feelings of depression, frustration, anxiety, anger, and irritability. In addition to mood swings, impulsivity, and anger quickly, physiological symptoms that can appear in changes in appetite, chest and back pain, muscle tension and teeth grinding, increased heart rate and blood pressure, sleep disturbance, and headaches^16^. As well as cognitive symptoms that affect the individual’s mind, such as negative thoughts, lack of concentration, and difficulty making decision and solving problems^17^.

### Causes for mental illness

The biopsychosocial model creates a holistic view to explore the biological, psychological, and social factors of your life.

- Biological factors, genetic characteristics and an individual’s brain chemistry can increase the risk of mental illness, especially if there is a family history of the illness^18^.
- Psychological factors, are related to the individual’s thoughts and beliefs, his vision of life, and his sense of the events around them.
- Social factors, which are the surrounding environment including work, relationships, social life, and culture can have influence on mental health^18^.

According to the WHO, “300 million people around the world suffer from mental illness”, which is equivalent to 3.4% of the world’s population, and that out of every group of five adults, one person suffers from mental illness^18^.

### Consequences of mental health

The consequences of mental disorders have a significant impact on the individual’s lifestyle^18^, which can be observed through various aspects of life as:

- Mental disorder has a significant impact on an individual’s family, as they must adapt to various symptoms that may include mood swings, losing interest of certain activities and other changes that may affect the family’s lifestyle. Moreover, they should be aware of the mental illness that the family member suffers from, monitor the development of symptoms, and follow up with a psychiatrist.
- Social isolation is a state in which an individual is isolated from the real world and lacks interaction with others which increases feelings of loneliness and the risk of mortality affecting the individual’s physical and mental health^19,20^.
- Happiness is an important state of mind for human life in general, and feeling happy is linked to enjoying life. Lack of happiness makes the individual does not have the energy to communicate or interact with people in any activity, it also affects the individual’s thoughts and their view of things. Happiness can decrease due to surrounding events or feelings and their impact on the individual’s mental health, as any mental illness has side effects such as feeling sad, depressed, anxious, and other feelings that have a major influence on happiness and enjoyment of life^21^.
- Academic life is affected by the psychological state of the student^22^. The impact of mental illness on a student’s academic life can be seen through his academic performance and grades, his relationship with his colleagues and doctors, and his participation in activities.
- It has been shown that there is a relationship between mental illness and violent thoughts and behaviors that lead to committing criminal acts. Although not all people with mental illness engage in criminal behavior, some mental illnesses have side effects that, along with other factors such as environment and childhood, may lead to an increased tendency towards violence and criminality^23^.
- According to the study conducted at the Australian rural mental health association^24^, 48% of people diagnosed with depression had suicidal thoughts while another 16% have made suicide attempts, this highlights the idea that people suffer from mental illness have high risk of suicidal thought.

The WHO with a collaboration with the Harvard School of Public Health, has created a comprehensive study^25^, which includes an analysis of the main causes of the burden of disease. In 1990, depressive disorder was ranked fourth out of fifteen leading causes of global disease burden, and in 2020, it reached the second rank of causes of the global burden of disease, due to the impact of the COVID-19 pandemic on the world and the people’s mental health^26^. For further notice, it is expected that by 2030, mental disorders as depression will be the leading cause of burden worldwide^27,28^.

### Artificial intelligence prediction of mental health disorders

AI technology has impacted mental healthcare in various ways such as collecting data about the patient through photos, videos, music they listen to, posts and interactions through social media, and information from wearable devices such as smart watches, after collecting these data, machine learning (ML) algorithms predict the individual’s mental health by analyzing data collected^29^. Also, after a patient is diagnosed with a mental illness, natural language processing (NLP) algorithms can be used to monitor patient’s behavioral changes which can be noticed in the patient’s language in conversations with other people in chats or emails, which indicates the effectiveness of treatment. Also, chatbots are expected to take over clinical visits to psychiatrics as it can detect mental illnesses and recommend different techniques to recover from them^30^.

### Contribution

The research focuses on utilizing different ML algorithms in mental healthcare for prediction of several mental illness. The contributions of the paper is summarized as follow:

- Comprehensive review of ML algorithms for mental illness prediction: A detailed review of existing ML techniques utilized in mental illness prediction is conducted, highlighting their strengths, limitations, and applicability in various clinical and research settings.
- Development of a ML-based predictive framework: A novel framework for mental illness prediction is proposed, leveraging multiple ML algorithms, including random forest (RF), decision tree (DT), naïve bayes (NB), k-nearest neighbors (KNN), and support vector machine (SVM), to enhance predictive accuracy and robustness.
- Integration of the DASS-42 questionnaire for model training and testing: The depression, anxiety, and stress scale (DASS-42) questionnaire is utilized as a validated psychometric tool for training and evaluating the proposed ML models, ensuring that predictions are based on established psychological assessment metrics.
- Comprehensive performance evaluation: The proposed framework is rigorously assessed using multiple performance metrics, including accuracy, confusion matrix, precision, recall, and F1-score, to ensure a comprehensive evaluation of its effectiveness in mental illness prediction.

Comprehensive review of ML algorithms for mental illness prediction: A detailed review of existing ML techniques utilized in mental illness prediction is conducted, highlighting their strengths, limitations, and applicability in various clinical and research settings. Development of a ML-based predictive framework: A novel framework for mental illness prediction is proposed, leveraging multiple ML algorithms, including random forest (RF), decision tree (DT), Naïve Bayes (NB), k-nearest neighbors (KNN), and support vector machine (SVM), to enhance predictive accuracy and robustness. Integration of the DASS-42 questionnaire for model training and testing: The depression, anxiety, and stress scale (DASS-42) questionnaire is utilized as a validated psychometric tool for training and evaluating the proposed ML models, ensuring that predictions are based on established psychological assessment metrics. Comprehensive performance evaluation: The proposed framework is rigorously assessed using multiple performance metrics, including accuracy, confusion matrix, precision, recall, and F1-score, to ensure a comprehensive evaluation of its effectiveness in mental illness prediction.

## Related work

Over the past years, research and studies have been conducted to benefit from AI technology in the field of mental health care. Mental health assessment and prediction using machine learning algorithms have gained substantial attention in recent years. In^31^, DASS21 questionnaire employed to predict depression, anxiety, and stress using multiple ML algorithms. For depression, their accuracies ranging from 61.21% using DT compared to 94.08% using their proposed scheme, which combined SVM with Adaboost. Similarly, for anxiety, the proposed scheme achieved an accuracy of 92.89%, outperforming individual models like NB, RF, and SVM with accuracy 56.38%, 65.21%, and 89.54%, respectively. Moreover, for stress prediction, their proposed scheme reached 93.49% which is significantly higher than the individual models, such as XGBoost and SVM with accuraacy 69.93% and 89.54%, respectively. However, their approach relies solely on the DASS21 questionnaire, which may introduce biases due to self-reporting limitations and may not generalize well across different demographic groups. Additionally, the effectiveness of the questionnaire for different cultural and linguistic backgrounds remains unverified.

In^32^, the authors developed their own questionnaire to predict mental health conditions. Using SVM, they achieved high accuracies of 96.15% for depression, 94% for PTSD, and 97.27% for anxiety, while logistic regression demonstrated exceptional accuracy for insomnia 98%. Furthermore, in^33^, Twitter API data were utilized to analyze social network mental disorders (SNMDs). Among the classifiers employed, their proposed SVM-CNN followed by long short-term memory (LSTM) system achieved the highest accuracy of 83.76%, surpassing RF with 82.2% accuracy, and DT with 79.3% accuracy.

In^34^, DASS21 questionnaire was used to assess anxiety, depression, and stress. For anxiety, DT and NB achieved the highest accuracies of 73.3%, while for depression, NB 85.5% and RF 79.8% outperformed other models. Stress prediction results showed a similar trend, with NB 74.2% and RF 72.3% achieving the best performances. Likewise^12^ leveraged the DASS21 questionnaire to achieve high accuracies across multiple algorithms, with KNN standing out for stress 94.5%, anxiety 94.5%, and depression 97.7%.

In^35^, SVM was applied to clinical data for attention deficit hyperactivity disorder (ADHD) diagnosis, achieving 66.1% accuracy. The relatively low performance suggests that traditional ML models may struggle to capture the complexity of ADHD-related features, highlighting the need for more sophisticated feature engineering or multimodal data integration. Similarly^36^ utilized CNN-LSTM to analyze recorded videos for detecting bipolar disorder, with a reported accuracy of 63.32%. Despite leveraging deep learning, the model’s performance indicates potential limitations in video-based assessment, such as the variability in facial expressions, lighting conditions, and individual differences in behavior. Moreover^37^ applied ML to MRI images for schizophrenia detection, where deep learning achieved the highest accuracy 94.44%, followed by RF 83.33% and SVM 82.68%. While promising, MRI-based approaches are costly, require specialized equipment, and may not be feasible for large-scale mental health screening.

In^38^, evaluation ML algorithms, particularly SVM and logistic regression (LR), for predicting depression, anxiety, and stress severity based on the DASS-42 questionnaire. The results indicate that LR outperforms SVM after hyperparameter tuning, achieving accuracies of 98.15% for depression, 98.05% for anxiety, and 98.45% for stress. The study highlights the importance of tuning ML models for enhanced performance but is limited by its dataset, which lacks diversity across different localities.

In^39^, explores the impact of AI on mental health assessment, particularly in response to the COVID-19 pandemic. Using the DASS-42 dataset and demographic information, multiple ML classifiers, including SVM, DT, RF, AdaBoost, CatBoost, and XGBoost, are compared for predicting depression, anxiety, and stress levels. SVM achieves the best performance with F1-scores of 94% for depression, 95% for anxiety, and 91% for stress. It demonstrates the effectiveness of SVM but it does not explore deep learning techniques or alternative data sources beyond survey responses.

## System model

**Fig. 1:**
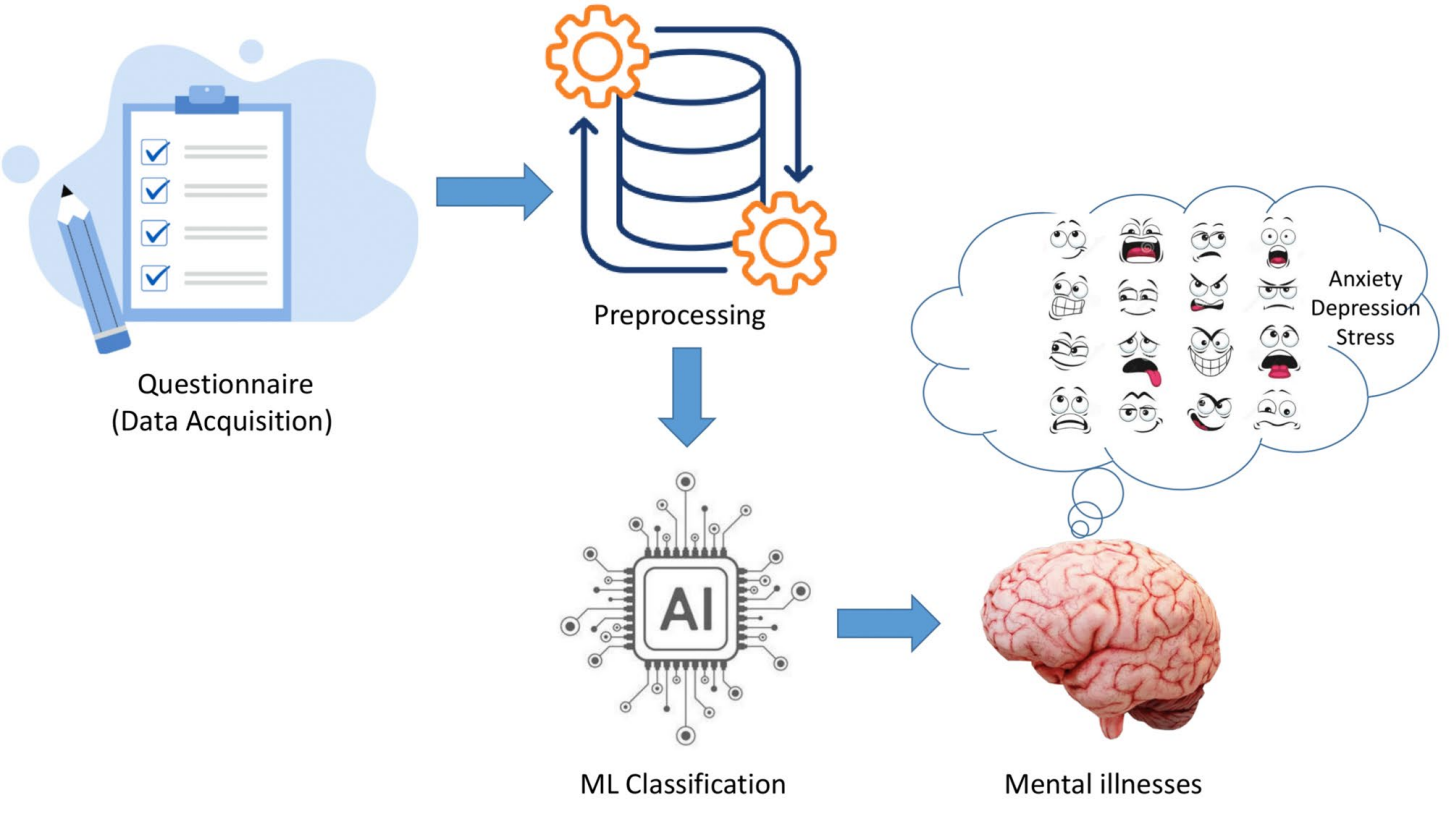
ML-based mental health assessment pipeline: This figure shows how questionnaire data is preprocessed and classified using machine learning to detect anxiety, depression, and stress.

The proposed scheme for mental illness detection is structured into several key phases, as depicted in Fig. 1. The system leverages ML techniques to classify individuals based on their responses to a standardized questionnaire or online survey. The model pipeline consists of data acquisition, preprocessing, and classification, which are described as follows.

### Data acquisition

Data is collected in real-time from participants who respond to a validated mental health assessment questionnaire. The questionnaire consists of a set of *N* questions, where each response $X_i$ (for $i = 1,2,\dots ,N$) is mapped to a numerical scale representing the participant’s mental state. The response vector for an individual can be represented as:

1 $$\begin{aligned} \textbf{X} = [X_1, X_2, \dots , X_N] \in \mathbb {R}^N \end{aligned}$$

### Data preprocessing

The dataset used in this study was obtained from Kaggle and includes approximately 39,775 anonymized responses collected between 2017 and 2019. The survey combined responses from the DASS-42 questionnaire and various demographic fields. Inclusion criteria included participants who completed all DASS-42 questions and submitted valid demographic metadata. Exclusion criteria involved removing responses with flagged meaningless words or those with significant missing data. For optional fields like “education,” missing values were replaced with a placeholder such as “No degree,” while essential fields resulted in case-wise deletion. The sample includes a variety of age and gender groups: 30,366 male, 8788 female, and 619 other respondents. Marital status distribution included 28,601 never married, 4265 currently married, and 1185 previously married participants. Educational backgrounds ranged from high school (15,634) to graduate degree holders (4066).

Before applying ML models, preprocessing is performed to enhance data quality and ensure consistency. The key preprocessing steps include:

- Handling missing data: Missing values in significant attributes are replaced with predefined placeholders, while rows with critical missing or infinite values are removed to maintain dataset integrity.
- Feature selection: Non-essential columns, such as auxiliary response fields, are discarded to focus on key attributes like DASS-42 scores.
- Standardization and renaming: Columns corresponding to personality traits are renamed for clarity, and categorical attributes such as academic majors and personality traits are standardized to a common format.
- Categorical data conversion: Some categorical attributes, such as age groups and mental illness levels, are converted into numerical formats to enhance machine learning model compatibility.

### Classification model

ML classifiers are trained to predict mental illness conditions based on the preprocessed input data. Several classification models are considered, including:

- RF: An ensemble of DT, where the prediction is based on majority voting across multiple trees.
- DT: A tree-based model that splits data recursively based on information gain (IG) or Gini impurity *G*.
- NB: A probabilistic classifier based on Bayes’ theorem.
- KNN: A distance-based classifier where the classification is based on the majority label among the *k*-nearest data points.
- SVM: A model that finds the optimal hyperplane separating classes.

A detailed illustration for ML algorithms is illustrated in Machine Learning Model Section.

### Prediction and interpretation

After training, the system classifies the responses of new participants into one of several mental health categories, such as normal, mild, moderate, severe, or extremely severe conditions. The final prediction is made based on the probability score $P(Y | \textbf{X})$, where the highest probability determines the classification outcome. The proposed system model analyzes different ML techniques to improve the accuracy of mental illness classification. Our approach optimally selects the best performing model while incorporating robust pre-processing techniques to enhance prediction reliability.

## Machine learning model

Algorithm selection plays a critical role in constructing an effective prediction model, as different algorithms employ different prediction techniques, resulting in varying performance and accuracy. Therefore, five ML algorithms are evaluated and a comparative analysis is performed based on the performance of these models on the same training data.

### Decision tree (DT)

A DT is a tree-structured algorithm commonly used for classification and regression tasks. Classifies instances in a dataset by recursively splitting the feature space based on feature values. Each internal node represents a feature and each leaf node corresponds to a class label or output value. The decision-making process begins at the root node, proceeds through intermediate nodes based on feature values, and ends at a leaf node, indicating the final prediction.

The tree-building process involves splitting the data set at each node according to a selected criterion, such as the Gini index or the information gain. These criteria help to measure the quality of a split, aiming to maximize the purity of the resulting subsets. For example:

- Gini index (*G*):
  2 $$\begin{aligned} G = 1 - \sum _{i=1}^{k} p_i^2 \end{aligned}$$
  where, $p_i$ is the proportion of instances belonging to class $i$, and $k$ is the total number of classes.
- Information gain (*IG*):
  3 $$\begin{aligned} IG(D, A) = H(D) - \sum _{v \in \text {Values}(A)} \frac{|D_v|}{|D|} H(D_v) \end{aligned}$$
  where $H(D)$ is the entropy of the dataset $D$, $H(D_v)$ is the entropy of the subset $D_v$ after splitting on feature $A$, and $|D_v|$ is the size of subset $D_v$, and $|D|$ is the size of the original dataset.

The recursive partitioning continues until a stopping criterion is met, such as reaching homogeneous leaf nodes, achieving a maximum tree depth, or satisfying a minimum information gain threshold. This ensures the tree is not overfitted to the training data.

### Random forest (RF)

RF algorithm is an ensemble learning method that constructs a collection of DT and combines their outputs to improve prediction accuracy. For classification tasks, RF outputs the class that receives the majority vote among all the trees. For regression tasks, it averages the predictions of the individual decision trees. RF prediction $\hat{y}$ that obtained by aggregating the predictions of individual decision trees:

4 $$\begin{aligned} \hat{y} = \frac{1}{T} \sum _{t=1}^{T} f_t(x) \end{aligned}$$

where $T$ is the total number of trees in the forest and $f_t(x)$ is the prediction of the $t$-th tree for instance $x$.

RF reduces the risk of overfitting by averaging the predictions of multiple trees, thereby improving generalization performance. It handles large datasets effectively and is robust to noise in the data due to its randomization process. Additionally, RF can provide insights into feature importance by measuring how much each feature contributes to reducing the splitting criteria across the trees.

### K-nearest neighbor (KNN)

KNN algorithm is a non-parametric, lazy learning algorithm that stores the entire training dataset for prediction. To classify a new instance, KNN identifies the $k$-nearest neighbors and assigns the class based on a majority vote among these neighbors. The distance between instances is typically calculated using the Euclidean distance:

5 $$\begin{aligned} \text {Euclidean Distance} = \sqrt{\sum _{i=1}^{n} (x_i - y_i)^2} \end{aligned}$$

where $x_i$ and $y_i$ are the feature values of instances $x$ and $y$, and $n$ is the number of features. KNN is simple, effective for small datasets, and relies on the choice of $k$ and the distance metric to achieve optimal performance.

### Naïve Bayes

Naïve Bayes is a probabilistic classifier based on Bayes’ theorem, assuming conditional independence between features. The posterior probability of a class $c$ given a feature vector $x$ is computed as:

6 $$\begin{aligned} P(c | x) = \frac{P(x | c) P(c)}{P(x)} \end{aligned}$$

where $P(c | x)$ is posterior probability of class $c$ given $x$, $P(x | c)$ is likelihood of $x$ given class $c$, $P(c)$ is prior probability of class $c$, and $P(x)$ is prior probability of $x$. The independence assumption simplifies the likelihood as:

7 $$\begin{aligned} P(x | c) = \prod _{i=1}^{n} P(x_i | c) \end{aligned}$$

where $x_i$ is the $i$-th feature and $n$ is the number of features. Naïve Bayes is computationally efficient and effective for high-dimensional data.

### Support vector machine (SVM)

SVM is a classification algorithm that identifies a hyperplane to separate classes in a high-dimensional feature space. SVM handles both linear and non-linear classification tasks using kernel functions. The objective of SVM is to maximize the margin between the hyperplane and the support vectors, which are the data points closest to the hyperplane.

The decision function for linear classification is given by:

8 $$\begin{aligned} f(x) = \text {sign}(w^T x + b) \end{aligned}$$

where $w$ is weight vector, $x$ is feature vector of a data point, $b$ is bias term, and $\text {sign}(\cdot )$ is determines the class label based on the sign of the linear combination. For non-linear classification, SVM uses a kernel function $K(x, x')$ to map data into a higher-dimensional space:

9 $$\begin{aligned} f(x) = \text {sign} \left( \sum _{i=1}^{n} \alpha _i y_i K(x_i, x) + b \right) \end{aligned}$$

where $\alpha _i$ is lagrange multipliers, $y_i$ is class labels of training data, and $K(x_i, x)$ is kernel function, such as radial basis function (RBF) or polynomial kernel.

## Proposed scheme for predicting mental illness

**Fig. 2:**
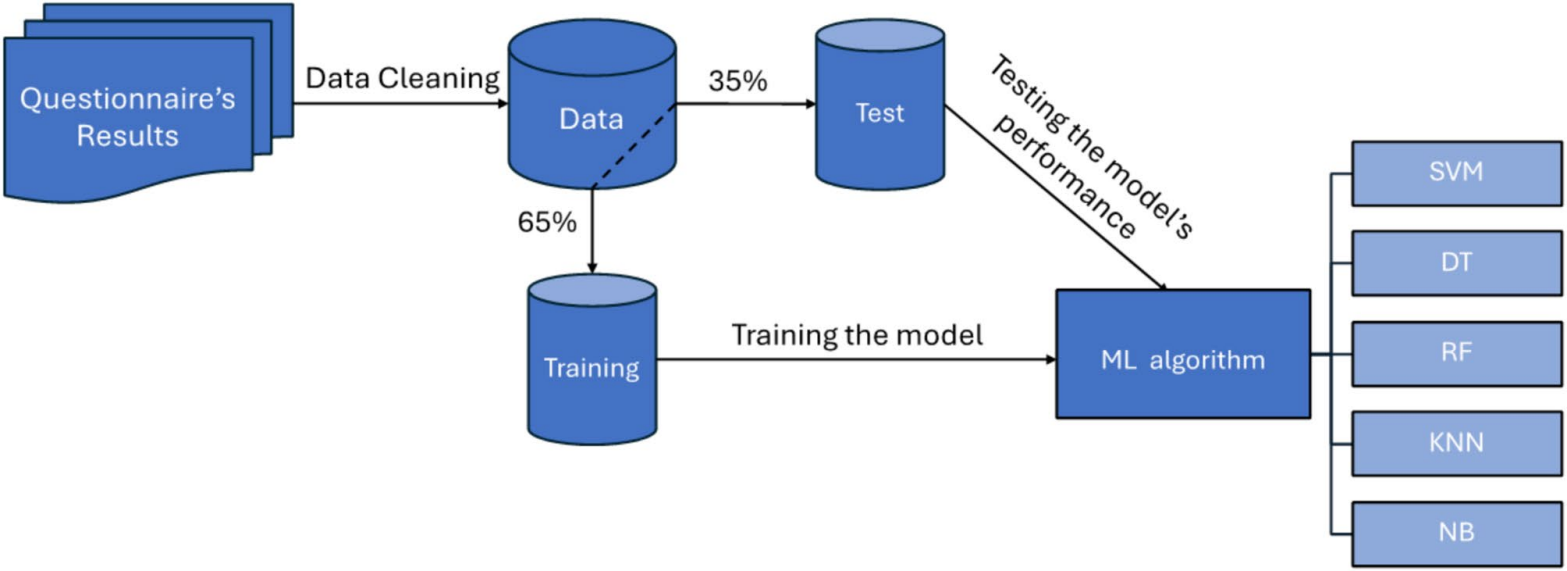
ML workflow for mental health prediction: The figure shows a pipeline where cleaned questionnaire data is split for training and testing ML models (SVM, DT, RF, KNN, NB) to classify mental health conditions like depression, anxiety, etc.

Predictive analysis leverages historical data to forecast future outcomes. In mental healthcare, timely diagnosis is crucial, as delays can significantly impact lives. ML enables rapid and precise diagnostics, addressing inefficiencies in traditional psychiatric evaluations and improving patient outcomes. One of the most widely used methods for diagnosing mental illness today involves questionnaires, which are employed by psychiatrists in clinics, schools, universities, and other organizations. ML models can enhance the accuracy of these diagnostic processes by analyzing data collected from questionnaire responses and delivering results more quickly than human evaluators.

ML classification algorithms, in particular, have shown great promise for predicting mental illness based on questionnaire results. This approach offers a practical and accessible solution for individuals who cannot afford psychiatric care. Additionally, it benefits those who may fear revealing their identity or opening up to others, as they might trust a machine over a human due to concerns about stigma, work, or reputation. The proposed scheme builds upon utilizing ML models to predict mental illness effectively and efficiently ensuring accessible and trustworthy mental healthcare for a broader population.

### Model architecture

The model utilizes supervised classification ML algorithms to predict the mental health conditions. These algorithms are designed to work with labeled datasets. The proposed scheme is shown in Fig. 2. It aims to predict three distinct mental health conditions, depression, anxiety, and stress disorder. This prediction is accomplished by utilizing data from the depression anxiety stress scales-42 (DASS-42) questionnaire.

The datasets is then split into two subsets, one for training ML models and the other for testing their performance. The training subset feeds into an ML algorithm module, where various algorithms, such as support vector machines (SVM), decision trees (DT), random forests (RF), k-nearest neighbors (KNN), and Naïve Bayes (NB), are applied^40^.

**Table 1:** Data format.

Case	Feature 1	Feature 2	$\cdots$	Feature *n*	Class
1	xxx	x	$\cdots$	xx	good
2	xxx	x	$\cdots$	xx	good
3	xxx	x	$\cdots$	xx	bad
$\vdots$	$\vdots$	$\vdots$	$\cdots$	$\vdots$	$\vdots$

These algorithms process the training data to identify patterns and relationships. Once trained, the models are evaluated using the test dataset to determine their accuracy, reliability, and suitability for making predictions or classifications. The model’s performance is subsequently evaluated based on its ability to accurately predict the severity of these mental health conditions based on the questionnaire’s results. This flow ensures that the chosen ML model is robust and well-validated before deployment. Table 1 shows a sample of the data format supervised classification algorithms deal with.

### Sample dataset of DASS-42

The DASS-42 questionnaire consists of 42 items assessing depression, anxiety, and stress on a 4-point Likert scale (0 = Did not apply to me at all, 3 = Applied to me very much or most of the time). Below are five illustrative items:

- I felt that life was meaningless. (Depression)
- I experienced trembling (e.g., in the hands). (Anxiety)
- I found it hard to wind down. (Stress)
- I felt that I was using a lot of nervous energy. (Stress)
- I felt I wasn’t worth much as a person. (Depression)

These sample items illustrate the type of structured input used for training machine learning models.

### Data collection

DASS-42 questionnaire is an updated version of the DASS-21, which has demonstrated efficacy in clinical settings. The DASS-42 is a self-report tool consisting of 42 items designed to assess the severity of depression, anxiety, and stress in individuals. This questionnaire is widely used in clinical practice due to the overlap in symptoms across these three conditions, making it challenging for both clinicians and patients to distinguish between them. The scores derived from the 42 items provide insight into the specific mental health challenges faced by the individual^41^.

Each of the three mental health conditions, depression, anxiety, and stress, has a dedicated set of questions that help identify the severity of the condition. The scores corresponding to each condition indicate the degree to which the individual is affected, as outlined in Table 2. The severity of each condition is scored based on a subset of the questionnaire items, with the total score for each condition defined as:

10 $$\begin{aligned} \text {Score}_{\text {Condition}} = \sum _{i \in Q_{\text {Condition}}} x_i \end{aligned}$$

where $Q_{\text {Condition}}$ represents the indices of the questions related to the specific condition, and $x_i$ is the response to the *i*-th question.

The dataset used for training the model is derived from a large survey containing responses to the DASS-42 questionnaire, along with additional demographic information (e.g., gender, age, etc.). The dataset, which spans responses from 2017 to 2019, is available on Kaggle. This dataset includes approximately 40,000 responses and encompasses several types of data:

- DASS-42 responses: The responses are categorized into three columns for each of the 42 questions:
  - Q1A to Q42A: Scores for the DASS-42 questionnaire items.
  - Q1E to Q42E: The time taken by participants to answer each question.
  - Q1I to Q42I: The position of each question on the participant’s screen.
- Ten item personality inventory (TIPI): This is a self-report scale that measures the big five personality traits, openness to experience, conscientiousness, extraversion, agreeableness, and neuroticism. It includes 10 items, each reflecting a dichotomous trait (e.g., extraverted vs. introverted), and is scored on a 7-point scale ranging from disagree strongly to agree strongly.
- Validity checklist (VCL): This component assesses the validity of the participant’s responses by checking for consistency and coherence in their answers. Certain words, identified as meaningless, are used to filter out invalid responses (e.g., cuivocal, florted, Verdid). If any of these words are flagged, the corresponding survey is excluded.
- Additional demographic information: The dataset includes demographic information such as education, age, gender, marital status, and other lifestyle-related questions, which are useful for contextualizing the results and visualizing the data.

**Table 2:** Score ranges for depression, anxiety, and stress.

	Depression	Anxiety	Stress
Normal	0–9	0–7	0–14
Mild	10–13	8–9	15–18
Moderate	14–20	10–14	19–25
Severe	21–27	15–19	26–33
Extremely severe	28+	20+	34+

### Data pre-processing

Data pre-processing is a crucial step in preparing the dataset for the ML model. This process helps to clean, transform, and structure the data to ensure optimal performance during training. The pre-processing steps are tailored to the specific characteristics of the dataset and are designed to reduce unnecessary complexity.

#### Steps in data pre-processing

- Handling missing data: Missing values in major attributes are replaced with “No degree”. Rows with missing or infinite values are removed to maintain the integrity of the dataset.
- Column removal: Non-essential columns, such as Q1E to Q42E and Q1I to Q42I , are discarded. The focus is placed solely on the DASS-42 scores (Q1A to Q42A).
- Renaming TIPI Columns: The columns corresponding to the TIPI are renamed to reflect the corresponding personality traits..
- Standardization of attributes: For example, participants may report their major using various terms (e.g., “Business Administration,” “Management,” “HR”). A function is applied to standardize these entries to a common format, ensuring consistency across the dataset.
- Categorical data conversion: Some categorical columns, such as age group and mental illness level, are converted to numerical formats to facilitate processing by ML algorithms. The age groups are clustered into five categories (e.g., “under 10,” “primary children,” “adults,” etc.).

To ensure valid performance measurement, we used a 70/30 stratified train/test split and employed 5-fold cross-validation on the training set. This method helps assess model generalization while ensuring that no test data influenced the training phase.

#### Data shape post-processing

Before pre-processing, the dataset contains 39,775 columns and 172 rows. After pre-processing, the dataset is refined, resulting in 68 rows, with unnecessary columns removed and missing values appropriately handled. This reduction in data dimensions ensures that the model is trained with relevant and clean data.

Given that certain fields like academic major were entered as open-text, we implemented a rule-based standardization algorithm using string normalization and Levenshtein distance matching. This allowed us to map similar responses (e.g., “Business Administration,” “Management”) to a consistent label. This preprocessing step helped preserve data integrity and improve model accuracy.

### Mental illness subsets and labeling

For each mental illness (depression, anxiety, and stress), subsets of the dataset are created based on the relevant DASS-42 questions. These subsets focus only on the items that contribute to the score for each condition. A new column, totalCount, is added to each subset, representing the total score for that condition. The totalCount is then mapped to a categorical condition column, which denotes the severity of the mental illness according to the scoring guide in Table 2. The levels of mental illness are categorized as normal, mild, moderate, severe, and extremely severe. This categorical data is subsequently transformed into numerical values to ensure compatibility with ML algorithms.

### Model training and evaluation

The dataset is split into two subsets: Training data 70% and testing data 30%. The training data is used to train the ML model, while the testing data is reserved to evaluate the model’s predictive accuracy. The pre-processed dataset, which now contains relevant features for predicting depression, anxiety, and stress, is ready for the ML. The model is trained on the training data and tested on the testing data to determine its ability to predict the mental health conditions accurately.

The ML algorithms utilize supervised learning to map the input data *X* (features from the questionnaire) to target labels *Y* (mental health conditions). The mapping is learned by minimizing a loss function $L(\hat{Y}, Y)$, where $\hat{Y}$ represents the predicted labels. For example:

11 $$\begin{aligned} L(\hat{Y}, Y) = \frac{1}{n} \sum _{i=1}^{n} \ell (y_i, \hat{y}_i) \end{aligned}$$

where $n$ is the number of data points, and $\ell$ is the cross-entropy loss function, which is used in all models for classification tasks.

## Evaluation of the classification model

The validation of a ML models relies on a set of evaluation metrics that measure its performance and effectiveness^42,43^. Several metrics are used to evaluate the classification performance of the model, including accuracy, precision, recall, and the F1-score.

### Accuracy

The accuracy of a model is the percentage of correct predictions made by the model out of all the predictions^44^. It is calculated as follows:

12 $$\begin{aligned} \text {Accuracy} = \frac{\text {Number of correct predictions}}{\text {Total number of predictions}} \end{aligned}$$

#### Confusion matrix

A confusion matrix is a table that visualizes the performance of a classification algorithm by comparing predicted and actual classifications^45,46^. The matrix contains the following values:

- True positives (TP): The number of instances correctly predicted as positive.
- True negatives (TN): The number of instances correctly predicted as negative.
- False positives (FP): The number of instances incorrectly predicted as positive (Type I error).
- False negatives (FN): The number of instances incorrectly predicted as negative (Type II error).

#### Precision

Precision indicates the proportion of true positive predictions among all positive predictions. It is calculated as:

13 $$\begin{aligned} \text {Precision} = \frac{\text {TP}}{\text {TP} + \text {FP}} \end{aligned}$$

#### Recall

Recall, also known as sensitivity, indicates the proportion of true positive predictions among all actual positive instances. It is calculated as:

14 $$\begin{aligned} \text {Recall} = \frac{\text {TP}}{\text {TP} + \text {FN}} \end{aligned}$$

#### F1-score

The F1-score is the harmonic mean of precision and recall, providing a balance between the two. It is calculated as:

15 $$\begin{aligned} \text {F1-Score} = \frac{2 \times \text {Recall} \times \text {Precision}}{\text {Recall} + \text {Precision}} \end{aligned}$$

## Simulation results

The proposed scheme utilize DASS-42 questionnaire for evaluation. Predicting of depression, anxiety, and stress illness is achieved using RF, DT, NB, KNN, and SVM algorithm’s. A comparison is made between the performance of five different machine learning classification algorithms to predict the level of three mental illnesses by using the results of DASS-42 questionnaire results. The data collected for the model contains 39,773 responses after data pre-processing. The count of people participated in the survey in terms of their gender is 30,366 male, 8788 female, and 619 others. Moreover, the count of people in terms of their marital status is 28,601 never married, 4265 currently married, and 1185 previously married. Furthermore, the count of people participated in the survey in terms of their age group is 16,188 secpndary children, 15,388 adults, 4960 primary children, 2081 elder adults, and 1156 older adults. The count of people participated in the survey in terms of their educational level is 15,634 high school, 15,065 less than high school, 5008 university degree, and 4066 graduate degree.

**Fig. 3:**
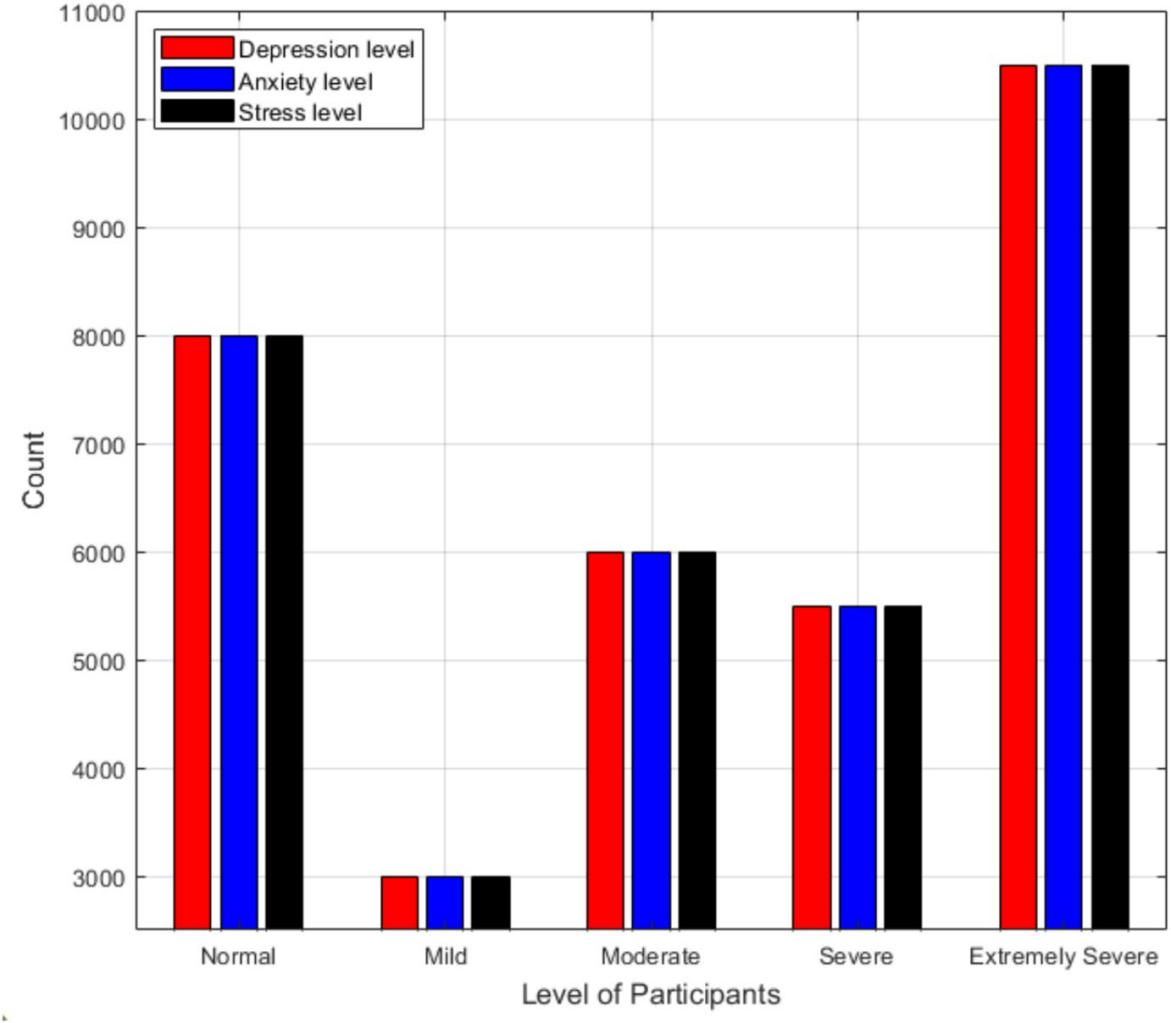
Depression, anxiety, and stress level of participants.

Figure 3 shows normal, mild, moderate, severe, and extremely severe for depression, anxiety, and stress illness. As shown, most of people suffering extremely severe for depression, anxiety, and stress. On the other hand mild condition counts the lowest number of people followed by sever then moderate condition then normal.

### Confusion matrices

**Fig. 4:**
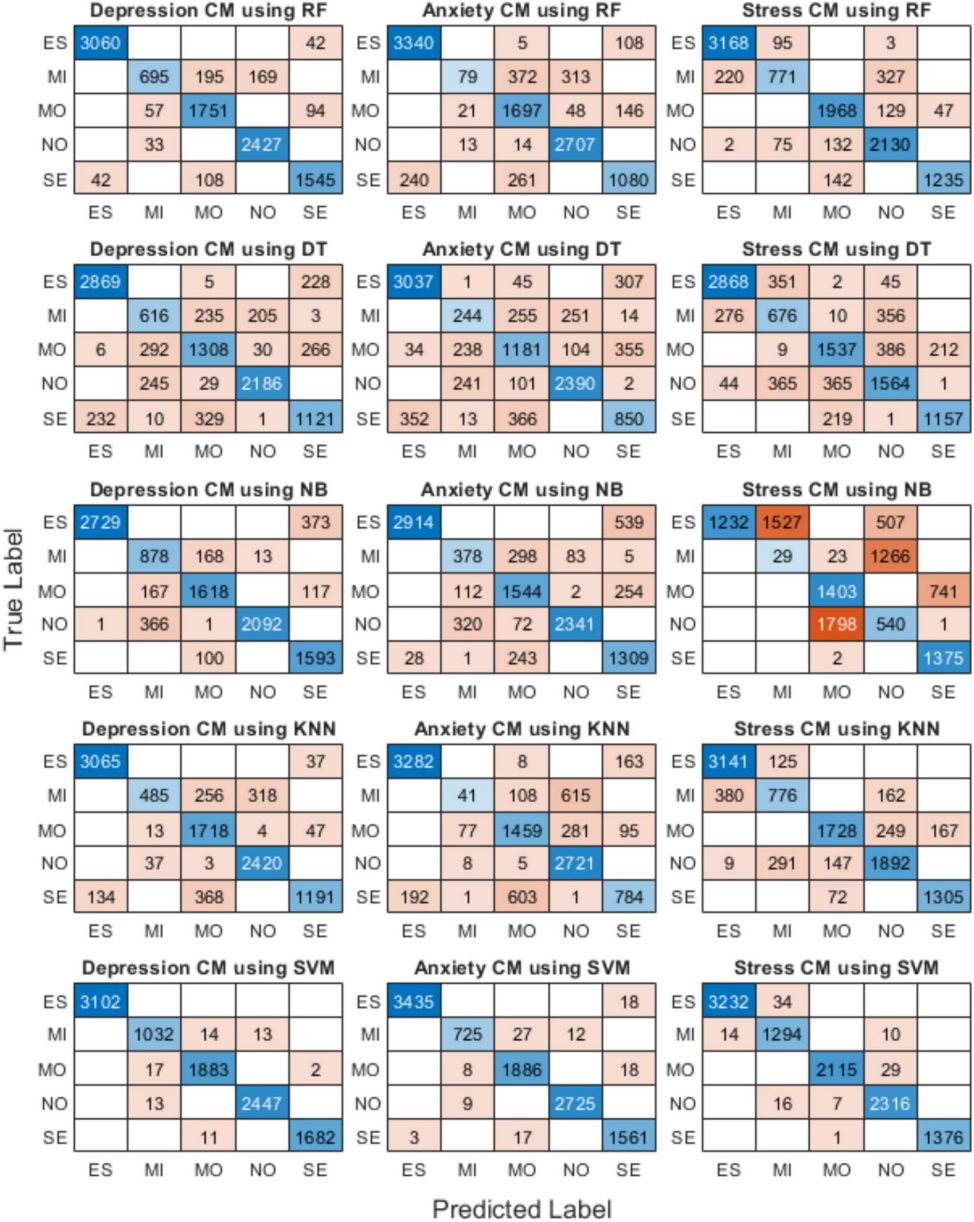
Confusion matrix for depression using RF, DT, NB, KNN, and SVM algorithms, for anxiety using RF, DT, NB, KNN, and SVM algorithms, and for stress using RF, DT, NB, KNN, and SVM algorithms. Note that: ES, MI, MO, NO, and SE stands for extremely severe, mild, moderate, normal, and severe, respectively.

Figure 4 shows the confusion matrix for depression using RF, DT, NB, KNN, and SVM algorithms, for anxiety using RF, DT, NB, KNN, and SVM algorithms, and for stress using RF, DT, NB, KNN, and SVM algorithms. Results shows that SVM stood out as the most accurate, achieving 97.1% for depression, 98.3% for anxiety, and 97.5% for stress. This means that SVM was highly effective at correctly categorizing different severity levels, from mild to extremely severe. Its strength lies in how well it can separate different cases by finding the best decision boundaries, even in complex datasets. Because of this, SVM consistently provided the best precision and recall, making it the most reliable model in this comparison.

Another strong performer was RF model, which reached 96.5% accuracy for depression, 92.7% for anxiety, and 94.8% for stress. RF’s success comes from its ability to handle a variety of cases without overfitting. It performed well across different severity levels and was especially good at recognizing extreme cases. However, it didn’t always match SVM’s performance in mid-range classifications, where distinguishing between mild and moderate symptoms was slightly more challenging. The KNN model also did fairly well, coming close to RF in accuracy. It was particularly effective at identifying the most extreme cases. However, KNN relies on distance-based calculations, meaning its performance depends heavily on how well the data is structured. It needed careful tuning to get the best results, and in some cases, it struggled when symptoms were too similar across categories.

On the other hand, DT model had a noticeable drop in accuracy, with 91.2% for depression, 89.6% for anxiety, and 88.2% for stress. It worked well when cases were clearly distinct, but it had difficulty classifying cases where symptoms overlapped. DT models tend to overfit, meaning they can perform very well on training data but struggle with new, unseen cases. This made them less reliable for nuanced classifications, though they remain useful in situations where explainability is a priority. The weakest model in this comparison was NB, which had 87.4% accuracy for depression, 84.3% for anxiety, and only 76.5% for stress. NB makes strong assumptions about the independence of features, which isn’t always realistic in mental health data. Because of this, it frequently misclassified mild and moderate cases. It was also particularly weak in stress prediction, suggesting that it wasn’t able to capture the subtle patterns necessary for accurate classification.

Overall, the results show that SVM is the best model for predicting depression, anxiety, and stress, offering the highest accuracy and best performance across different severity levels. Random Forest comes in as a strong second choice, especially in cases where interpretability and efficiency are important. KNN also showed promise but required fine-tuning to perform optimally. Decision Tree models, while decent, struggled with more ambiguous cases, and Naïve Bayes performed the worst due to its inability to handle complex relationships in the data. If further improvements were needed, exploring deep learning models or hybrid approaches could help refine predictions, especially in distinguishing between borderline cases.

**Table 3:** Evaluation metrics for depression, anxiety, and stress using RF, DT, NB, KNN, and SVM.

Metric	Algorithm	Depression	Anxiety	Stress
Accuracy	RF	92.8%	85.2%	88.8%
	DT	79.3%	73.7%	74.7%
	NB	87.2%	81.3%	85.2%
	KNN	86.9%	79.3%	84.7%
	SVM	99.3%	98.9%	98.8%
F1_Score	RF	92.6%	82.9%	88.4%
	DT	79.4%	73.7%	74.9%
	NB	87.7%	82%	85.7%
	KNN	86.2%	76.5%	84.4%
	SVM	99.3%	98.9%	98.8%
Recall	RF	92.8%	84.5%	88.8%
	DT	79.4%	73.7%	74.7%
	NB	87.2%	83.9%	85.2%
	KNN	86.9%	76.9%	84.7%
	SVM	99.3%	98.9%	98.8%
Precision	RF	92.8%	85.2%	87.8%
	DT	79.4%	73.75%	75%
	NB	89.4%	81.3%	87%
	KNN	87.1%	79.3%	84.3%
	SVM	99.3%	98.9%	98.8%

**Table 4:** Generalization gap analysis for depression, anxiety, and stress classification using SVM.

Generalization gap	Depression (SVM)	Anxiety (SVM)	Stress (SVM)
Training accuracy	0.999	0.997	0.991
Testing accuracy	0.993	0.989	0.989
Generalization gap	0.006	0.008	0.002
Result	The model is not overfitting	The model is not overfitting	The model is not overfitting

### Evaluation metrics

Table 3 shows evaluation metrics for depression, anxiety, and stress using RF, DT, NB, KNN, and SVM. Results reveals distinct performance variations across accuracy, F1-score, recall, and precision metrics. SVM outperforms all other classifiers in every category, achieving accuracy of 99.3% for depression, 98.9% for anxiety, and 98.8% for stress. Additionally, its F1-score, recall, and precision maintain similarly high values, reinforcing its reliability as a predictive model for mental health disorders. This demonstrates that SVM is highly effective in distinguishing between different mental health states and minimizing misclassification.

RF classifier performs well, with the second-best results, with accuracy scores of 92.8% for depression, 85.2% for anxiety, and 88.8% for stress. Its F1-score and recall closely match its accuracy, indicating a balanced performance. The RF model shows a robust ability to detect patterns in mental health data, making it a competitive alternative to SVM with slightly lower performance, particularly in anxiety classification.

NB classifier exhibits moderate effectiveness, with accuracy values of 87.2% for depression, 81.3% for anxiety, and 85.2% for stress. While its precision for depression is relatively high 89.4%, it struggles slightly in anxiety detection, as shown by its lower recall score 83.9%. Despite its simplicity, NB remains a viable option, particularly in cases where computational efficiency is a priority.

KNN algorithm follows a similar trend to NB, with accuracy scores of 86.9% for depression, 79.3% for anxiety, and 84.7% for stress. Although KNN provides reasonable predictions, its F1-score and recall are lower than those of RF and SVM, indicating that it may not be as reliable, especially for anxiety detection where it achieves the lowest recall at 76.9%. This suggests that KNN may struggle with differentiating borderline cases, leading to increased misclassification.

DT classifier ranks the lowest in overall performance, yielding accuracy scores of 79.3% for depression, 73.7% for anxiety, and 74.7% for stress. Its F1-score, recall, and precision mirror these results, indicating that while DT can still function as a predictive model, it lacks the robustness and generalization capabilities of more sophisticated classifiers like SVM and RF. The relatively poor performance suggests that DT models may overfit to training data, reducing their effectiveness in real-world applications.

**Fig. 5:**
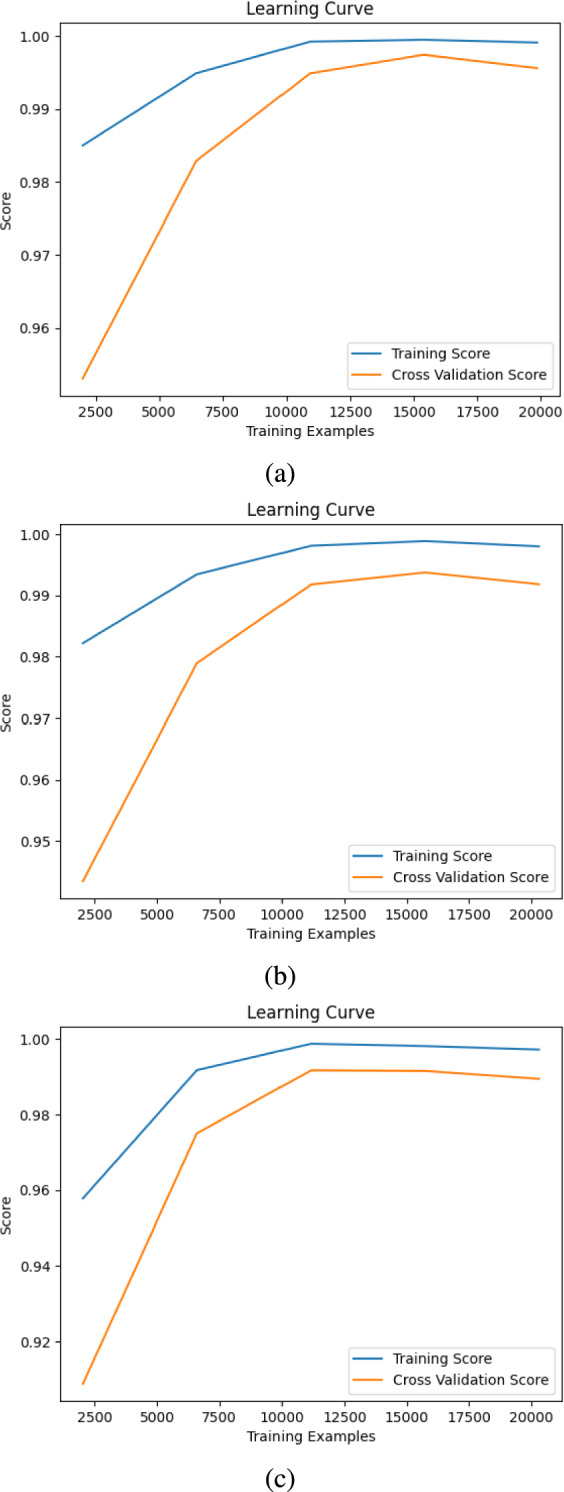
(**a**) Depression, (**b**) anxiety, and (**c**) stress learning curve using SVM.

### Overfitting evaluation

Cross-validation is a widely adopted and robust technique for assessing the performance of machine learning models. It involves partitioning the dataset into multiple subsets, commonly referred to as k-folds. During each iteration of the cross-validation process, one fold is reserved as the test set while the remaining k–1 folds serve as the training set. This iterative process ensures that the model is evaluated on various subsets of the data, preventing biases that may arise from using a single training or test set. Consequently, it provides a more reliable estimate of the model’s ability to generalize to new, unseen data.

In the present analysis, cross-validation is employed to evaluate the model’s performance, demonstrating that the model generalizes well across different data subsets without overfitting. The model’s accuracy is computed for each fold, and the average accuracy from all k iterations is taken as the final metric for performance. The results from this evaluation indicate that the model maintains consistent accuracy across the different folds, with no substantial discrepancies observed between the training and testing accuracies, thereby suggesting the absence of overfitting.

The generalization gap is an essential metric for identifying overfitting in machine learning models given by:

16 $$\begin{aligned} \text {Generalization Gap} = \text {Training Accuracy} - \text {Testing Accuracy} \end{aligned}$$

To evaluate overfitting, the generalization gap is compared to a threshold. If the gap exceeds 0.1, the model is considered to be overfitting. Otherwise, if the gap is smaller than 0.1, the model is not overfitting:

17 $$\begin{aligned} \text {Generalization Gap} = {\left\{ \begin{array}{ll} \text {Not Overfitting}, & \text {if } x < 0.1 \\ \text {Overfitting}, & \text {if } x \ge 0.1 \end{array}\right. } \end{aligned}$$

Table 4 shows generalization gap and model performance for depression, anxiety, and stress using SVM. Since all the generalization gaps are significantly below the 0.1 threshold, it can be confidently concluded that the models for depression, anxiety, and stress are not overfitting. The training and testing accuracies are very close to each other, which demonstrates that the models are generalizing well and can be expected to perform reliably on unseen data.

### Learning curve analysis

The learning curve is a widely used method to visualize the model’s learning performance across different training set sizes. It provides insights into whether a model is underfitting or overfitting^47^. An ideal model should exhibit two key characteristics: (1) a minimal gap between training and validation performance and (2) a non-decreasing validation curve as training data increases.

Figure 5 shows depression, anxiety, and stress learning curve using SVM. Depression Model (SVM) In Fig. 5a, the training score remains high across all training sizes, converging near 1.00. Meanwhile, the cross-validation score shows an initial sharp increase, showing little improvement beyond 12,500 training examples. The small gap between the training and validation scores suggests that the model generalizes well, with minimal overfitting.

In Fig. 5b, the cross-validation score starts lower but improves rapidly as more training data is introduced, eventually stabilizing. The final validation score approaches the training score, indicating that the model benefits from additional data without significant overfitting.

In Fig. 5c follows the same pattern observed in the depression and anxiety models. The validation score increases with more training data and stabilizes beyond 12,500 samples. The gap between training and validation performance remains small, further confirming that the model is well-regularized and exhibits strong generalization capabilities.

**Fig. 6:**
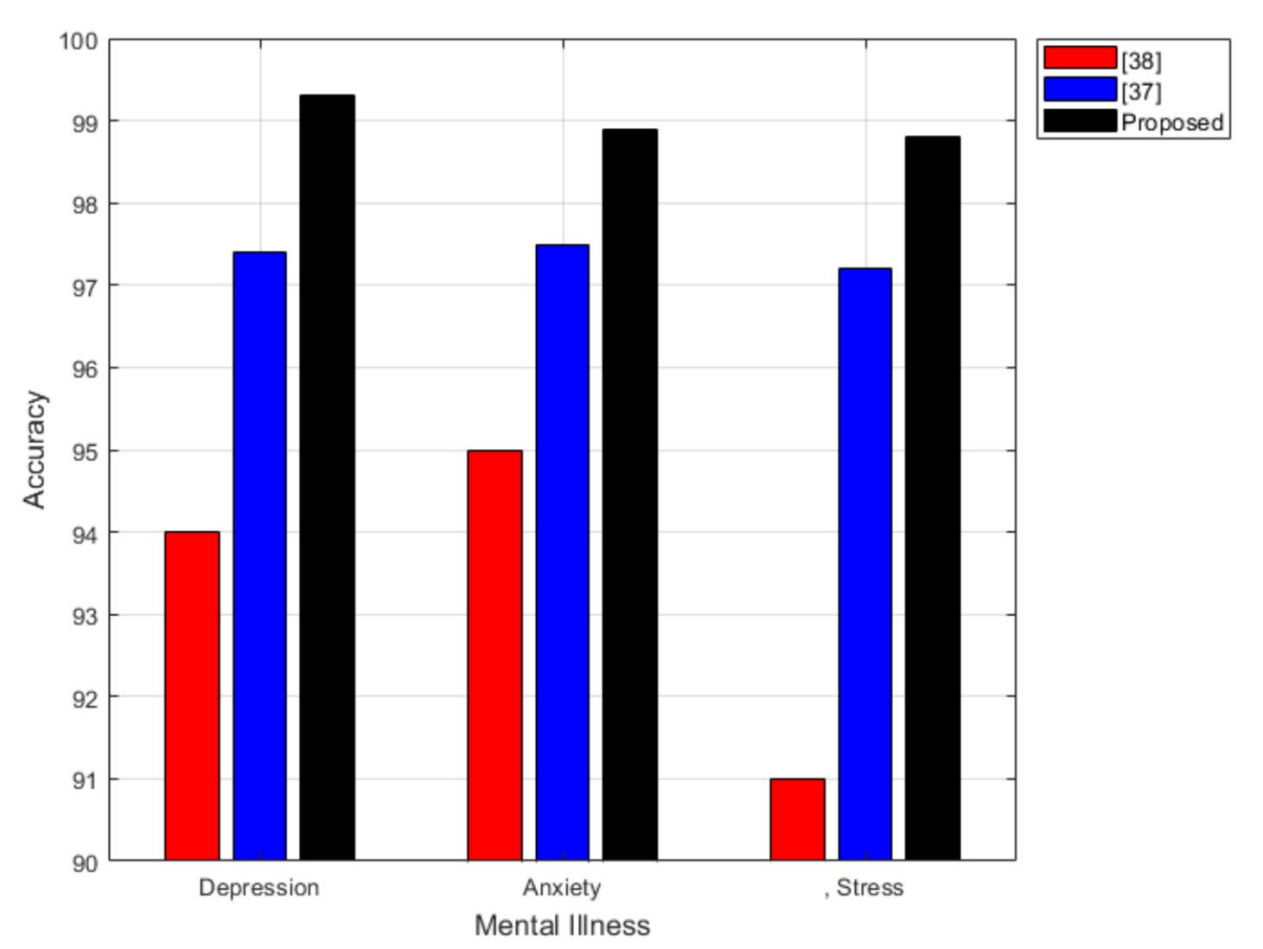
Compare the accuracy of the proposed scheme with^38^ and^39^ for predicting depression, anxiety, and stress accuracy using SVM for DASS-42 questionnaire.

The learning curves for all three models indicate that the SVM classifiers achieve high accuracy while maintaining generalization. The decreasing gap between training and validation performance with increasing data suggests that the models are not significantly overfitting. However, beyond 12,500 training examples, performance gains become marginal, suggesting a potential saturation point in model learning. Further improvements may require hyperparameter optimization, feature selection, or alternative ML architectures rather than simply increasing data.

### Comparison

The comparison in Fig. 6 clearly demonstrates the superior performance of the proposed scheme over previous works^38^ and^39^ in predicting depression, anxiety, and stress using SVM on the DASS-42 questionnaire. Notably, our approach achieves an accuracy of 99.3% for depression, 98.9% for anxiety, and 98.8% for stress, consistently outperforming both prior studies. While^38^ reports high accuracy, with a peak of 97.49% for anxiety, our model further refines the classification process, yielding an improvement of approximately 1.4–2.0% across all categories. Similarly, compared to^39^, which attains relatively lower accuracy (especially in stress prediction at 91%), our proposed scheme exhibits a substantial enhancement of nearly 8%. The observed improvements can be attributed to the advanced preprocessing techniques, optimized feature selection, and fine-tuned hyperparameters employed in our approach. By effectively leveraging these optimizations, our scheme reduces classification errors and enhances model generalization, making it a more reliable tool for mental health assessment.

### Discussion and limitations

This study provides evidence that traditional machine learning algorithms, particularly support vector machines, can accurately predict psychological distress based on questionnaire data. However, there are limitations to consider. First, the use of self-reported survey responses introduces potential bias, as these are subjective and may lack clinical validation. Second, demographic skews within the dataset (e.g., age or region representation) may affect generalizability. Lastly, open-text fields required additional preprocessing, which may introduce standardization errors. Future research may explore deep learning approaches and integrate multimodal data sources like voice or physiological signals. Validating these models in clinical settings would also strengthen their applicability in real-world healthcare environments. The predictive ability of AI-based models such as SVM highlights their potential role in real-time clinical settings. These models could serve as decision-support tools for clinicians, enabling rapid triage, early detection, and continuous monitoring of mental health conditions. For example, integration into telehealth platforms or mobile applications could allow patients to complete DASS-42 questionnaires remotely, with immediate risk stratification provided to clinicians. However, before deployment, clinical validation testing in healthcare environments is essential to ensure reliability, safety, and ethical application.

## Conclusion

Mental health is a crucial yet often neglected aspect of well-being. This study highlights the potential of machine learning (ML) in providing accessible and effective mental health support. By utilizing the depression anxiety stress scales-42 (DASS-42) questionnaire, we explored the capability of various ML algorithms—decision tree (DT), random forest (RF), k-nearest neighbor (KNN), naive Bayes (NB), and support vector machine (SVM)—to diagnose depression, anxiety, and stress disorders. SVM exhibited superior performance, achieving the highest accuracy across all three disorders 99.3% for depression, 98.9% for anxiety, and 98.8% for stress. RF also performed well, providing a competitive alternative with slightly lower accuracy. KNN showed reasonable predictive capability but required careful tuning to optimize results. DT and NB, while useful, exhibited lower accuracy and struggled with nuanced classifications, making them less reliable for complex mental health diagnoses.

## Data Availability

The dataset analyzed during the current study is publicly available on Kaggle at: https://www.kaggle.com/datasets/lucasgreenwell/depression-anxiety-stress-scales-responses/data?select=demo1.png.
